# Supplementary material for: EnzML: multi-label prediction of enzyme classes using InterPro signatures
Source: BMC Bioinformatics. 2012 Apr 25;13:61. doi: 10.1186/1471-2105-13-61 (PMC3483700; doi:10.1186/1471-2105-13-61)
Supplement: Addtional file 5 — The Java code to format the data files, evaluate and predict. The file enzml_java_code.tar.gz contains the Java code used to format database data to ARFF and XML formats, to execute cross and train-test (jackknife) evaluations and to record evaluation results to database. More information is included in the readme.txt file and the Javadoc files. The code can be used with a MySQL database. To use a different database software, other JDBC drivers might be required. [file 1471-2105-13-61-S5.gz › java_code/utils/doc/uk/ac/ed/inf/utils/database/DbWriter.html]

DbWriter


---


|  |  |  |
| --- | --- | --- |
| **Overview** \| **Package** \| **Class** \| **Use** \| **Tree** \| **Deprecated** \| **Index** \| **Help** | |  |
| **PREV CLASS**   **NEXT CLASS** | **FRAMES**    **NO FRAMES**     **All Classes** |  |
| SUMMARY: NESTED \| FIELD \| CONSTR \| METHOD | DETAIL: FIELD \| CONSTR \| METHOD |  |


---


## uk.ac.ed.inf.utils.database Class DbWriter

```
java.lang.Object
  uk.ac.ed.inf.utils.database.DbManaged
      uk.ac.ed.inf.utils.database.DbWriter
```

---

``` public class DbWriter extends DbManaged ```

To write to a database table

**Version:**
:   20 Feb 2009

**Author:**
:   Luna De Ferrari luna.deferrari-at-ed.ac.uk

---

**Constructor Summary**

`DbWriter(DbManager dbManager)`


| **Method Summary** | |
| --- | --- |
| `java.sql.ResultSet` | `executeQuery(java.lang.String sqlQuery)`             Executes any SQL update which returns some results. |
| `java.lang.Integer` | `executeQueryReturn1Id(java.lang.String sqlInsertInto)`             Executes an SQL update (an individual row insert) and returns the auto-generated key (identifier) of the first inserted row. |
| `java.sql.ResultSet` | `executeQueryReturnIds(java.lang.String sqlInsertInto)`             Executes an SQL update (usually a row insert) which returns the identifier(s) of the inserted rows. |
| `boolean` | `executeUpdate(java.lang.String sqlToExecute)`             Executes any SQL update which does not return results (create/drop table etc) |
| `TableWriter` | `getTableWriter(java.lang.String table)`             Object to write to a table |

**Methods inherited from class uk.ac.ed.inf.utils.database.DbManaged**

`getDbConnection, getDbManager`

**Methods inherited from class java.lang.Object**

`equals, getClass, hashCode, notify, notifyAll, toString, wait, wait, wait`

**Constructor Detail**

### DbWriter

```
public DbWriter(DbManager dbManager)
```


**Method Detail**

### executeQuery

```
public java.sql.ResultSet executeQuery(java.lang.String sqlQuery)
```

:   Executes any SQL update which returns some results.

    :   **Parameters:**: `sqlQuery` - sql code to execute **Returns:**: the results

---


### executeQueryReturn1Id

```
public java.lang.Integer executeQueryReturn1Id(java.lang.String sqlInsertInto)
```

:   Executes an SQL update (an individual row insert) and returns the
    auto-generated key (identifier) of the first inserted row.

    :   **Parameters:**: `sqlInsertInto` - sql code to execute **Returns:**: the auto-generated key of the first inserted row

---


### executeQueryReturnIds

```
public java.sql.ResultSet executeQueryReturnIds(java.lang.String sqlInsertInto)
```

:   Executes an SQL update (usually a row insert) which returns the
    identifier(s) of the inserted rows.

    :   **Parameters:**: `sqlInsertInto` - sql code to execute **Returns:**: the identifiers of the inserted rows

---


### executeUpdate

```
public boolean executeUpdate(java.lang.String sqlToExecute)
```

:   Executes any SQL update which does not return results (create/drop table
    etc)

    :   **Parameters:**: `sqlToExecute` - sql code to execute **Returns:**: true if the sql command was executed

---


### getTableWriter

```
public TableWriter getTableWriter(java.lang.String table)
```

:   Object to write to a table


---


|  |  |  |
| --- | --- | --- |
| **Overview** \| **Package** \| **Class** \| **Use** \| **Tree** \| **Deprecated** \| **Index** \| **Help** | |  |
| **PREV CLASS**   **NEXT CLASS** | **FRAMES**    **NO FRAMES**     **All Classes** |  |
| SUMMARY: NESTED \| FIELD \| CONSTR \| METHOD | DETAIL: FIELD \| CONSTR \| METHOD |  |


---
